# Inflammatory markers in children with obstructive sleep apnea syndrome

**DOI:** 10.3389/fped.2023.1134678

**Published:** 2023-04-11

**Authors:** Yingge Wang, Ying Chen, Wei Lin, Min Huang, Yuanteng Xu, Guohao Chen

**Affiliations:** ^1^ Department of Otorhinolaryngology, The First Affiliated Hospital, Fujian Medical University, Fuzhou, China; ^2^ Department of Sleep Medicine Center, National Regional Medical Center, Binhai Campus of the First Affiliated Hospital, Fujian Medical University, Fuzhou, China; ^3^ Fujian Institute of Otorhinolaryngology, The First Affiliated Hospital, Fujian Medical University, Fuzhou, China; ^4^ The Second Affiliated Hospital of Xiamen Medical College, Xiaman, China

**Keywords:** YKL-40, inflammatory factors, interleukin, OSA (Obstructive sleep apnea), obstructive sleep apnea syndrome

## Abstract

**Objective:**

To evaluate serum inflammatory markers of YKL-40, Interleukin-6 (IL-6), Interleukin-8(IL-8), Interleukin-10(IL-10), TNF-α(tumor necrosis factor-α), and CRP (C-reactive protein) in children with and without OSAS.

**Methods:**

The ELISA technique has been used to identify the concentration of inflammatory markers such as YKL-40, IL-6, IL-8, IL-10, TNF-α, and CRP in the serum of 83 children with OSAS and 83 children without OSAS.

**Results:**

Serum levels of YKL-40, IL-6, IL-8, and IL-10 were found to be increased in children with OSAS. YKL-40 was found to be positively correlated with IL-6 and IL-8, and negatively correlated with IL-10. At the same time,YKL-40 was also found to be positively correlated with OAHI and LoSpO2% in OSAS group. IL-8 was positively correlated with OAHI whereas IL-10 was positively correlated with LoSpO2.

**Conclusion:**

Children with OSAS are in a systemic inflammatory state. YKL-40 together with IL-8 may act as serum inflammatory markers and provide an indication for the diagnosis of children with OSAS.

## 1. Introduction

Obstructive sleep apnea syndrome (OSAS) has become one of the common diseases affecting 1.2% to 5.7% of children. Symptoms of pediatric OSAS include habitual sleep snoring, mouth breathing, repeated awakening, enuresis, excessive sweating, hyperactivity, and so on (1, 2). Severe OSAS causes associated complications such as growth retardation, cardiovascular disease, neurocognitive abnormalities, behavioral problems, and even causes craniofacial malformations and thoracic deformity (3, 4). Studies showed that children with OSAS caused greater burdens on family finance and quality of life than children without OSAS at all ages (5).

At present, polysomnography (PSG) is still the gold standard for diagnosing OSAS in children. Due to poor coordination, some snoring children cannot be successfully diagnosed as OSAS by PSG. To simplify the diagnosis of pediatric OSAS, there is a great necessity to identify serum biomarkers which can be used as surrogates of PSG. A growing number of studies depict that pediatric OSAS is associated with a systemic inflammatory response. Inflammatory markers such as IL-6, IL-8, CRP, TNF-α, INF-γ, ICAM, and VCAM have been found to increase in children with OSAS in accordance with different reports (6–9). However, there are no specific serum biological markers available for the diagnosis of OSAS. In recent years, we have focused on an inflammatory factor, YKL-40, which is involved in other inflammatory diseases and can promote the elevation of some cytokines. YKL-40 has not been reported in pediatric OSAS so far. This study is to verify the systemic inflammation in children with OSAS and explore the specific inflammatory markers for pediatric OSAS.

## 2. Materials and methods

A total of 83 children aged from 2 to 14 years old who underwent adenotonsillectomy (ATE) for OSAS were recruited in our hospital from 2020 July to 2021 February.The diagnosis of OSAS was confirmed by PSG. The children were monitored at night for at least 7 h in a quiet and comfortable state by Alice 5 Philips sleep monitors. Physiologic parameters included finger oxygen saturation, nose and mouth airflow, respiratory effort, snoring, sleep stages, body position, and heart rhythm. The data was processed by computers and manually corrected by professional technicians. The criteria for pediatric OSAS was according to Chinese guideline for the diagnosis and treatment of childhood obstructive sleep apnea (2). Mild OSAS is obstructive apnea hypopnea index (OAHI) between 1 and 5 events per hour, moderate OSAS is OAHI >5 to ≤10 events per hour, and severe OSAS is OAHI greater than 10 events per hour. We screened 83 children by OSA-18 questionnaire and those with a score of below 19 (10) in their physical examination in our hospital were recruited as the control group according to 1:1 of matched age and gender during the same period. The research was approved by an Ethics Committee (IEC-FOM-013-1.0). Informed consent was obtained from the participants and their guardians when the serum was collected. Exclusion criteria were acute inflammation; respiratory tract infection or asthma; cardiac disease and obesity.

## 3. ELISA of inflammatory markers

Fasting peripheral blood samples of 2 ml were drawn from all children after awakening in the morning. The supernatant was taken by centrifugation at 3,000 r/min and stored in a frozen depository marked tube under −80°C temperature. The inflammatory mediators' concentrations of YKL-40, IL-6, IL-10, IL-8, TNF-α, and CRP were determined by ELISA kits (Invitrogen United States) according to the manufacturer's protocol. The sensitivity of YKL-40, IL-6, IL-8, IL-10, TNF-α, and CRP were 10.83 pg/ml;0.92 pg/ml; ≤5 pg/ml; 1.0% pg/ml; 2.3 pg/ml and ≤10 pg/ml respectively. Optical density at 450 nm was determined using an Auto-Reader Model (SpectraMax i3x, MD, CA).

## 4. Statistical analysis

Data are expressed using mean ± standard deviation or median and interquartile spacing. Statistical analyses is performed using the GraphPad Prism (version 7) statistical software. *χ*^2^ tests are used to compare categorical variables in different groups. Wilcoxon or Kruskal-Wallis rank sum tests are used to analyze significant differences between groups. Spearman rank correlation or Pearson is used to analyze the correlation. *P* value <0.05 is considered statistically significant.

## 5. Results

### 5.1. Demographic characteristics of children at baseline

A total of 83 children with OSAS which includes [54 (65%)] boys and [29 (35%)] girls with a mean age of 7.0 ± 2.7 years old compared with 83 children without OSAS including the same percentage of boys and girls with a mean age of 6.8 ± 3.5 years. The children's characteristics in the three subgroups categorized by OSAS severity at baseline are shown in Table 1. There was no significant difference in age and BMI between OSAS and control groups. There was no significant difference in the course of the disease, tonsil size, and adenoids' size among the three subgroups. Significant differences in OAHI and LoSaO2% were noted among the three subgroups of pediatric OSAS.

**Table 1:** Demographic characteristics in children at baseline.

Characteristic	OSAS (*n* = 83)	Normal (*n* = 83)	*P*-value	Mild-OSAS (*n* = 53)	Moderate-OSAS (*n* = 11)	Severe-OSAS (*n* = 19)	*P*-value
Age (Year)	7 ± 2.7	6.8 ± 3.5	0.425	6.9 ± 2.5	7.6 ± 2.8	6.8 ± 3.2	0.631
Sex (Male/Female)	54/29	54/29	–	31/22	7/4	16/3	0.130
BMI (kg/m^2^)	17.1 ± 3.3	16.9 ± 3.4	0.656	16.5 ± 3.0	17.7 ± 3.6	18.2 ± 3.8	0.306
Course of disease (Month)	27.5 ± 29.1	–	–	28.9 ± 29.8	29.2 ± 35.3	22.6 ± 23.8	0.740
Tonsils size (I/II/III)	8/39/36	–	–	4/24/25	2/7/2	2/8/9	0.358
Adenoid size (A/N)	0.7 ± 0.1	–	–	0.7 ± 0.1	0.7 ± 0.1	0.7 ± 0.1	0.527
OAHI	7.9 ± 8.4	–	–	3.8 ± 2.4	5.6 ± 4.0	13.5 ± 11.0	**<0** **.** **001**
LoSpO2%	89.6 ± 4.5	–	–	91.4 ± 3.2	89.9 ± 2.0	84.3. ± 5.3	**<0**.**001**

Age, Year; Sex, Male/Female; BMI, body mass index; Course of disease, Month; Tonsils size: Degree of I–IV; Normal: control group; OAHI, obstructive apnea hypopnea index; LoSpO2%, lowest oxyhemoglobin saturation by pulse oximetry.

The bold value indicates a statistical significance.

### 5.2. Inflammatory markers in children with and without OSAS

The levels of YKL-40, IL-6, IL-8, and IL-10 in the serum of children were higher in the OSAS group than in the control group, and a significant difference (*P* < 0.05) was noted between the two groups. The levels of CRP and TNF-α were higher in OSAS than in the control group, but there was no significant difference (*P *> 0.05) between the two groups (Table 2).

**Table 2:** Inflammatory markers in children with and without OSAS.

	OSAS (*n* = 83)		Normal (*n* = 83)		*P*-value
	M	P_25_–P_75_	M	P_25_–P_75_	
**YKL-40 (ng/ml)**	29.28	22.98–39.32	25.13	20.79–32.84	**0**.**027**
CRP (mg/L)	0.19	0–1.39	0.03	0.01–0.31	0.566
**IL-6 (pg/ml)**	1.91	0–2.63	0.00	0–1.73	**0**.**002**
TNF-α (pg/ml)	830.22	616.20–996.52	813.46	626.5–975.72	0.761
**IL-8 (pg/ml)**	66.65	40.19–121.42	28.29	15.21–56.79	**<0**.**001**
**IL-10 (pg/ml)**	7.075	2.48–12.79	4.86	0–8.53	**0**.**008**

M, median (IQR); P_25_–P_75_, M (P25, P75).

The bold value indicates a statistical significance.

### 5.3. Inflammatory markers in subgroups of OSAS

**Figure 1:**
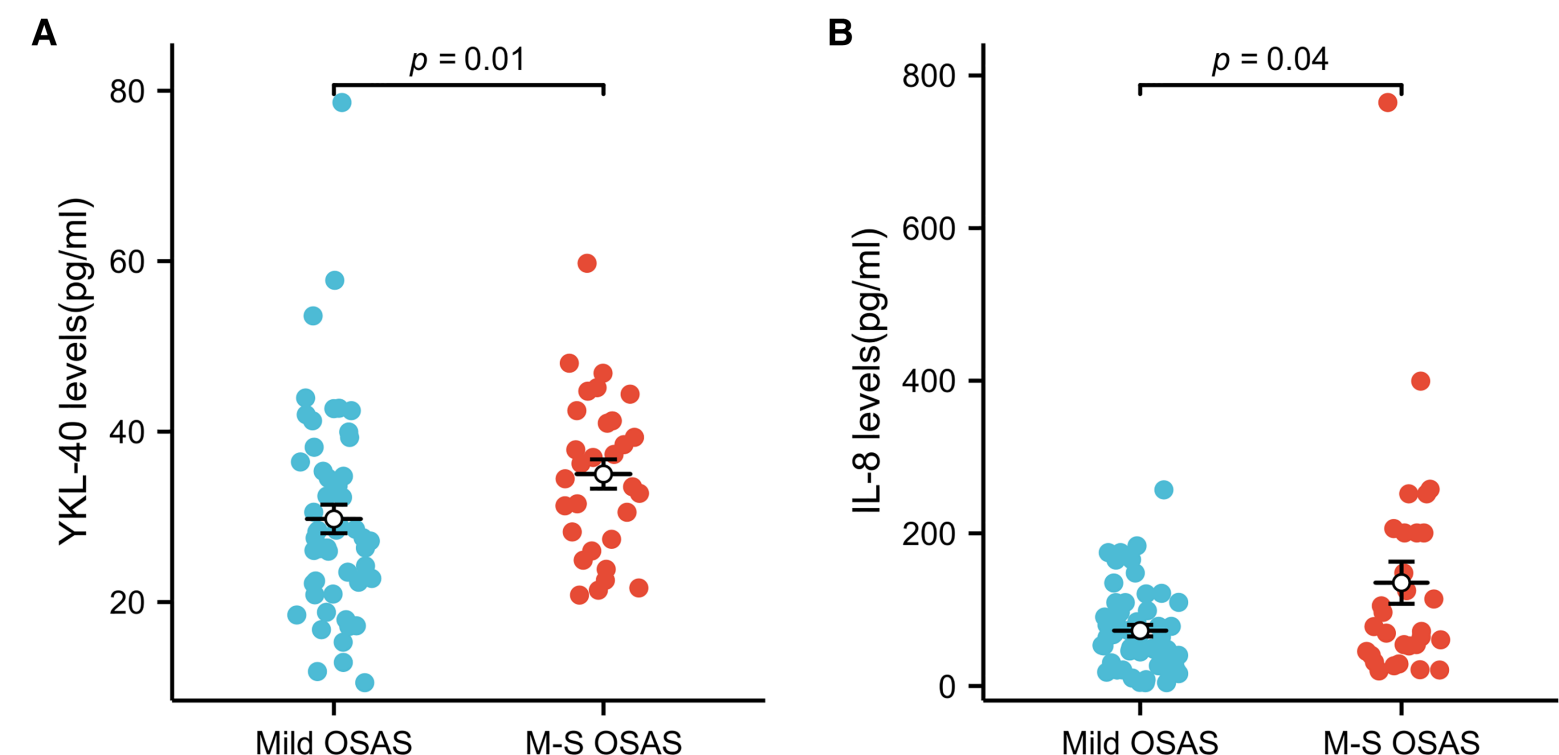
YKL-40 and IL-8 level in subgroups of OSAS. (**A**) Serum levels of YKL-40 in mild OSAS and moderate and severe groups (**B**) serum levels of IL-8 in mild OSAS and moderate and severe groups.

Serum levels of YKL-40 were higher in the severe than moderate OSAS group and moderate OSAS than mild OSAS group. But there was no significant difference among the three groups (*P *> 0.05). There was no significant difference in the levels of IL-8, IL-10, and TNF-α among the three groups (*P* > 0.05). The level of IL-6 was highest in the moderate group and a significant difference was noted among the three groups (*P* < 0.05). The level of CRP was highest in the severe group compared with the mild and moderate OSAS groups (*P* < 0.05) (Table 3). Combine moderate and severe OSAS, Serum levels of YKL-40 and IL-8 was found statistical differences between mild and moderate-severe groups (Figure 1).

**Table 3:** Inflammatory markers in subgroups of OSAS.

Characteristic	Mild-OSAS (*n* = 53)		Moderate-OSAS (*n* = 11)		Severe-OSAS (*n* = 19)		*P*-value
	M	P_25_–P_75_	M	P_25_–P_75_	M	P_25_–P_75_	
YKL-40(ng/ml)	28.41	22.77–36.44	31.53	27.37–36.97	37.87	23.85–44.4	0.267
**CRP (mg/l)**	0.00	0.00–1.01	1.13	0.19–1.52	1.30	0.16–1.72	**<0**.**001**
**IL-6(pg/ml)**	0.435	0.00–2.17	2.63	2.05–3.38	2.24	1.74–2.61	**<0**.**001**
TNF-α (pg/ml)	857.77	693.7–985.58	775.92	93.17–1,058.51	1,032.69	899.63–1,121.34	0.053
IL-8 (pg/ml)	68.22	42.405–115.12	54.14	28.87–113.99	71.51	45.27–148.06	0.757
IL-10 (pg/ml)	8.36	3.36–12.55	6.57	1.99–15.12	5.75	0–13.98	0.872

M, median (IQR); P_25_–P_75_, M (P25,P75).

The bold value indicates a statistical significance.

### 5.4. Correlation between the inflammatory factors and the baseline data in OSAS

YKL-40 was positively correlated with OAHI and LoSpO2% in OSAS group (*P* < 0.05). CRP was positively correlated with adenoid size. IL-8 was found to be positively correlated with OAHI in the OSAS group (*P* < 0.05) whereasIL-10 was positively correlated with LoSpO2% (*P* < 0.01) (Table 4, Figure 3).

**Table 4:** Correlation between the inflammatory factors and the baseline data.

	Course of disease (Month)	Tonsils size (I/II/III)	Adenoid size (A/N)	OAHI	LoSpO2
	*P*-value	*P*-value	*P*-value	*P*-value	*P*-value
YKL-40	0.465	0.622	0.632	**0.038 (*r* = 0.295)**	**0.018 (*r* = −0.33)**
CRP	0.594	0.566	0.154	0.107	0.066
IL-6	0.607	0.154	0.993	0.619	0.481
TNF-α	0.878	0.751	0.614	0.287	0.629
IL-8	0.051	0.770	0.296	**0.043 (*r* = 0.287)**	0.299
IL-10	0.431	0.350	0.382	0.353	**0.006 (*r* = 0.289)**

OAHI, obstructive apnea hypopnea index; LoSpO2%, lowest oxyhemoglobin saturation by pulse oximetry.

The bold value indicates a statistical significance.

### 5.5. Correlation among the inflammatory factors

In OSAS group, the correlation among the inflammatory factors was analysed.

**Figure 2:**
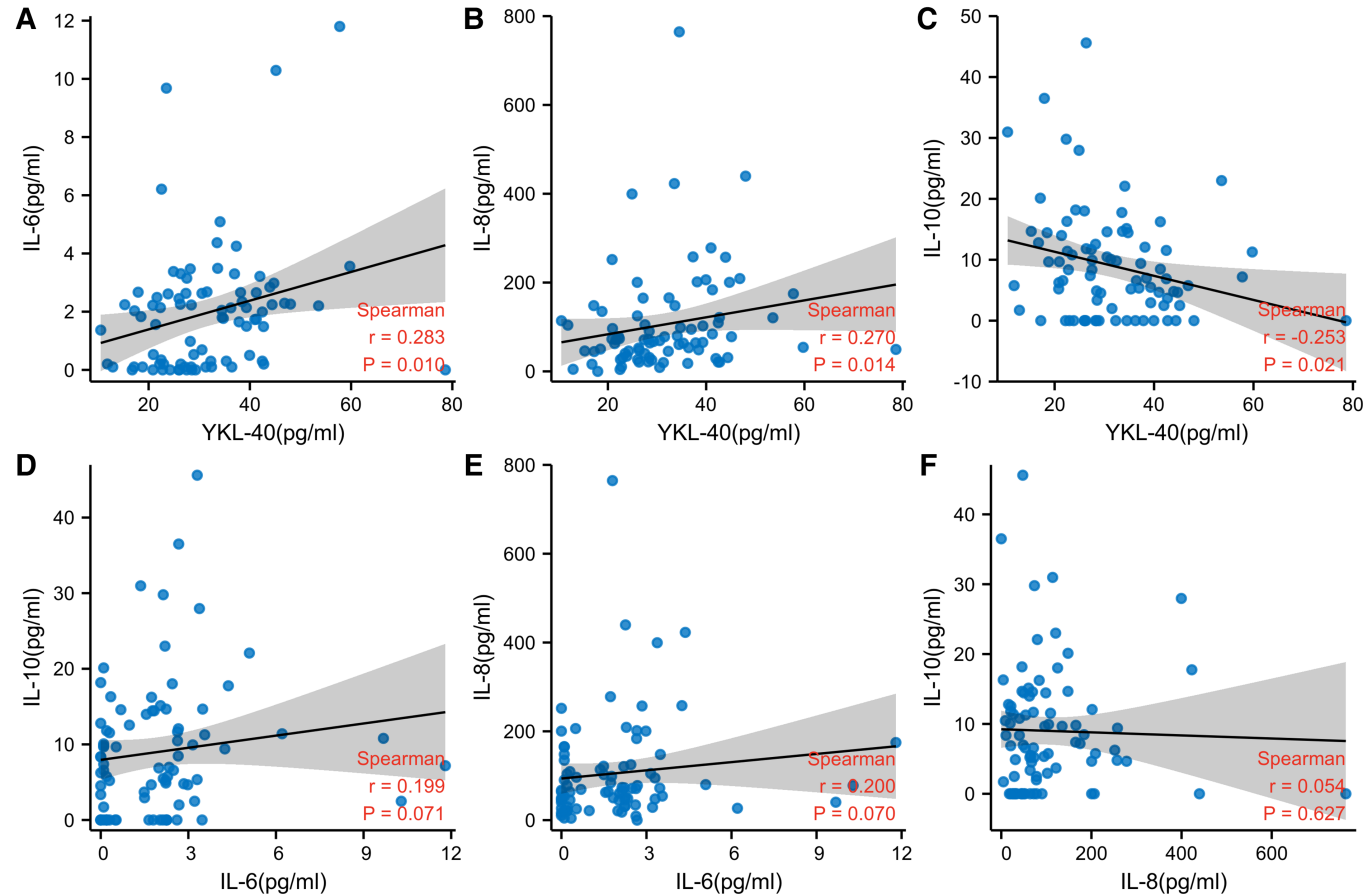
Correlation between the inflammatory factors. (**A**) Spearman correlation between YKL-40 and IL-6 (**B**) spearman correlation between YKL-40 and IL-8 (**C**) spearman correlation between YKL-40 and IL-10 (**D**) spearman correlation between IL-6 and IL-10 (**E**) spearman correlation between IL-6 and IL-8 (**F**) spearman correlation between IL-8 and IL-10. (weak correlation: 0.1 < *r* < 0.3, moderate correlation: 0.3 < *r* < 0.5, strong correlation: 0.5 < *r* < 1.0).

YKL-40 was positively correlated with IL-6 and IL-8 (*P* < 0.05). YKL-40 was negatively correlated with IL-10 (*P* < 0.05). There were no correlations between IL-6 and IL-8, IL-6 and IL-10, IL-8 and IL-10 (*P* > 0.05) (Figure 2).

### 5.6. ROC curves of the inflammatory markers for OSAS

**Figure 3:**
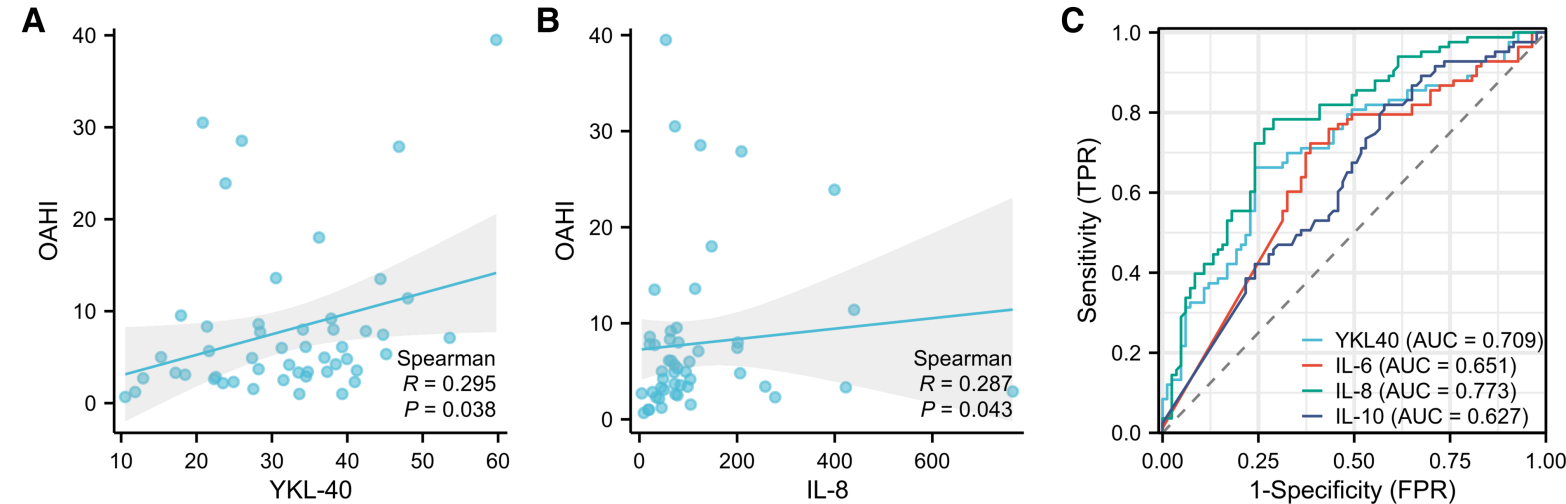
Correlation between YKL-40, IL-8, and OAHI, ROC curves of the inflammatory markers for OSAS. (**A**) Correlation between YKL-40 and OAHI (**B**) correlation between IL-8 and OAHI (**C**) area Under Curve (AUC) of YKL-40, IL-6, IL08, IL-10. (0.5–0.7 AUC shows low accuracy, 0.7–0.9 AUC shows accuracy, and above 0.9 AUC shows high accuracy).

In order to evaluate the predictive value of these inflammatory markers for OSAS, YKL-40, IL-6, IL-8, and IL-10 which had significant differences between OSAS and control group were selected to operate characteristic ROC (receiver operating characteristic) curve. AUC (Area Under Curve) was used for the evaluation of diagnostic tests and AUC >0.5 can be used as a valid diagnosis (Figure 3). Cut-off values are shown in Table 5.

**Table 5:** Information of ROC.

	Cut-off value	Sensitivity	Specificity	Precision	Positive predictive value
YKL40	25.78	0.66	0.76	0.71	0.733
IL-6	1.49	0.72	0.62	0.67	0.652
IL-8	45.65	0.78	0.71	0.75	0.730
IL-10	9.60	0.82	0.42	0.62	0.586

## 6. Discussion

Chronic persistent mild inflammation has become the most likely pathogenesis factor to cause OSAS (6), and the degree of the inflammatory response is correlated with the severity of OSAS (9). Among the inflammatory markers in pediatric OSAS, serum concentrations of IL-6, IL-8, IL-10, TNF-α, and CRP were the most frequently reported, but the association of these inflammatory factors with OSAS varied in different studies.

Our results suggest that the levels of IL-6, IL-8, and IL-10 in the serum of children were higher in OSAS than in the control group, but the levels of TNF-α and CRP had no significant difference in the two groups. Our results were consistent with the study of Albert M Li et al. (9). They found children with OSAS had higher serum IL-6 and IL-8, but TNF-α had no difference between children with and without OSAS. In Tam's study, serum levels of IL-8 were elevated, but IL-6, IL-10, TNF-α, and CRP had no change in children with and without OSAS. Higher IL-6 and lower cytokine IL-10 in non-obese children with OSAS were reported in Gozal's study (6). These differences may be caused by different races, age distribution, the severity of OSAS, and different selection of the control group. In our study, the control group was composed of healthy children without sleep-related symptoms, but in other studies, the control group was selected by children with habitual snoring.

YKL-40 is a proinflammatory factor, which is secreted by macrophages, neutrophils, fibroblasts, hepatic stellate cells, endothelial cells, and epithelial cells. YKL-40 maintains the homeostasis of various organs and participates in inflammatory reactions (11). YKL-40 has been reported enhanced in adult OSAS, and its level was correlated with apnea-hypopnea index (AHI) in adults with OSAS (12, 13).

We found elevated YKL-40 levels in children with OSAS compared to the healthy control group. Moreover, in the study of subgroups, serum YKL-40 levels were higher in the severe than the moderate OSAS group and in the moderate than the mild OSAS group, but there was no significant difference among the three groups. The results were different from L.C. MUTLU's report in adults with OSAS. Maybe the uneven case distribution of patients and mild OSAS was predominant among the three groups, which has caused no significant difference in YKL-40 levels among the three groups.

When we combined moderate and severe, there were statistical differences between mild and moderate-severe groups. Positive correlation was noted between YKL-40 and OAHI, as well as between YKL-40 and LoSpO2 in our study. This demonstrates YKL-40 may be used as a potential possible biomarker for screening pediatric OSAS and an indicator of OSAS severity. This is the first report about serum YKL-40 levels in pediatric OSAS. The relationship between YKL-40 and OSAS needs further studies *in vitro* and *in vivo*.

In the study of IL-6, IL-8, IL-10, TNF-α, and CRP in subgroups of pediatric OSAS, IL-6 and CRP were foundto have significant differences among the three groups. We found the level of IL-6 was highest in the moderate group.Combined with clinical knowledge, this result may not be clinically significant. The bias which can occur in the result could be due to the uneven distribution of disease severity.

IL-6 is a proinflammatory cytokine and an initiator of the inflammatory response. Serum and plasma interleukin-6 levels were higher in OSAS compared to healthy controls (14). Under the condition of inflammation caused by injury, IL-6 is secreted by T cells and macrophages in the tonsils and induces the activation, proliferation, and differentiation of T cells to participate in the immune response of the body (15). Therefore, it is speculated that intermittent hypoxia in children with OSAS may cause cell damage in tissues and promote the secretion of IL-6 by T cells and macrophages.

CRP was also found to show a significant difference among the three groups. The finding was also consistent with many reports which has published previously (16–18).

The expression of CRP was IL-6 dependent and greatly regulated by IL-6 (18, 19). But we found no significant difference in CRP between the OSAS group and the control group nor a correlation between CRP and IL-6 in this study. Therefore, the expression and interaction of inflammatory factors in OSAS are more complex than those reported in the literature and studies at present IL-8, IL-10, and TNF-α had no differences in the subgroups. When the OSAS group was divided into two groups, IL-8 had a significant difference between the mild and moderate-severe groups. IL-8 was positively correlated with OAHI. So, IL-8 may be used as a marker for the prediction of OSAS severity.

On the correlation among other inflammatory factors, significant positive correlations were noted between YKL-40 and IL-6, YKL-40, and IL-8. Anegative correlation was noted between YKL-40 and IL-10. The increase of circulating YKL-40, IL-6, IL-8, and IL-10 and the association between YKL-40 and IL-6, IL-8, and IL-10 has been reported separately in different inflammatory diseases. In 2011, Anders R. Nielsen demonstrated that IL-6 had a key role in the regulation of plasma YKL-40 levels during inflammation (20). In 2014, Tuija Väänänen reported that the levels of YKL-40 were higher andits concentration was correlated positively with IL-6 in osteoarthritis (21). The relation between YKL-40 and IL-8 was reported in colitis-associated neoplasia (22) and asthma (23). The demonstrated YKL-40 has enhanced the secretion of IL-8 under inflammatory conditions and could be a useful biomarker for patients with neoplasia and asthma. In 2012, Appleby LJ reported CHI3L1 level was elevated and negatively associated with IL-10 in Schistosoma haematobium-infected children (24).

The associations among IL-6, IL-8, and IL-10 were were found to be differently depicted in different publishes. We could not find the exact correlations between IL-6 and IL-8, IL-6 and IL-10, and IL-8 and IL-10 in pediatric OSAS. This may be due to the existence of different control groups, different races, different age distribution, different disease severity, and so on. The correlation, interaction, and mechanism of inflammatory factors need to be thoroughly studied in the future.

Recently, a case-control study showed that osteoprotegerin, chitinase 3-like protein 1(YKL-40, AUC = 0.9734), and cardiotrophin-1 could be used as potential biomarkers of OSA in adults (25). The ROC curve analysis performed for the serum levels of YKL-40, IL-6, IL-8, and IL-10 in our study. The result showed YKL-40 and IL-8 (AUC > 0.7) had more accurate predictive capabilities for pediatric OSAS compared to IL-6 and IL-10 (AUC < 0.7). The cut-off value of serum levels of YKL-40 is 25.78 and IL-8 is 45.65. For pediatric OSAS prediction as a screening test, the concentrations of YKL-40 (>25.78 pg/ml) and/or IL-8 (>45.65 pg/ml) in serum could be utilized.

## 7. Conclusion

As a conclusion, the study of the influence of serum inflammatory biomarkers in children found that the serum levels of YKL-40 were increased in those with Obstructive Sleep Apnea Syndrome (OSAS). YKL-40 levels were directly correlated with OAHI and LoSpO2,and the serum IL-8 levels were correlated specifically with OAHI. YKL-40 and IL-8 were significantly different between the mild and moderate-severe groups.YKL-40 together with IL-8 found to be specific serum inflammatory factors and which may provide an indication for the diagnosis and prediction of severity in children with OSAS. The cut-off value of serum levels of YKL-40 and IL-8 could be used as a reference value for clinical diagnosis. The limitation of invention and study might be the uneven distribution and severe OSAS were relativelyminor and therefore inflammatory markers in subgroups of OSAS need further optimization.

## Data Availability

The original contributions presented in the study are included in the article/Supplementary Material, further inquiries can be directed to the corresponding authors.
